# Real-world outcomes of selective laser trabeculoplasty in a tertiary referral glaucoma service

**DOI:** 10.1007/s10792-025-03702-3

**Published:** 2025-07-31

**Authors:** David Gildea, Jeremy O’Connor, Aoife Doyle

**Affiliations:** Royal Victoria Eye & Ear Hospital, Adelaide Road, Dublin 2, Ireland

**Keywords:** Selective laser trabeculoplasty, SLT, Real world, Glaucoma, Ocular Hypertension

## Abstract

**Introduction:**

Selective laser trabeculoplasty (SLT) is an effective treatment option in open-angle glaucoma (OAG) and ocular hypertension (OHT). The purpose of this study was to evaluate the real-world treatment outcomes of SLT in a tertiary referral glaucoma service.

**Methods:**

We reviewed the medical records of consecutive patients who had undergone SLT in the glaucoma service at the Royal Victoria Eye and Ear Hospital, Dublin. All patients in this study were using topical IOP-lowering medications prior to SLT. The primary outcome measure was the mean change in intraocular pressure (IOP) at 6 to 8 weeks following the procedure.

**Results:**

71 eyes of 44 patients were included in this study. There was a mean reduction in IOP of 4.0 mmHg (SD 3.6), from 19.1 mmHg (SD 3.6) at baseline to 15.1 mmHg (SD 4.0) at 6 to 8 weeks post-SLT (p < 0.001). The diagnosis was primary open-angle glaucoma (POAG) in 69.0% of eyes, pseudoexfoliation glaucoma (PXFG) in 5.6%, pigment dispersion glaucoma (PDG) in 2.8%, normal tension glaucoma (NTG) in 5.6%, ocular hypertension (OHT) in 15.5%, and pseudoexfoliation OHT in 1.4%. 100% of eyes were on topical IOP-lowering medication (mean number of agents 2.7). In addition, 8.5% had one previous SLT procedure. No immediate (< 1 h) post-SLT IOP spikes occurred in this study. Pearson correlation analysis revealed a moderate correlation (r = -0.4021, p < 0.001) between higher baseline IOP and greater IOP reduction.

**Conclusion:**

SLT is an effective IOP-lowering treatment in open-angle glaucoma and ocular hypertension in a real-world setting.

## Introduction

Raised intraocular pressure (IOP) is the most important modifiable risk factor in open-angle glaucoma (OAG) and ocular hypertension (OHT) [1, 2] Topical IOP-lowering medications have been the mainstay of management traditionally [1, 2] More recently, selective laser trabeculoplasty (SLT) has become an alternative first-line treatment option in the management of OAG and OHT [3].

SLT is a relatively straightforward outpatient-based laser procedure that selectively targets the pigmented trabecular meshwork, resulting in improved aqueous outflow and lowering of IOP [3] It is a well-tolerated procedure and has a good safety profile, with the benefit of not requiring daily compliance with IOP-lowering drops [3] The LiGHT trial was a landmark trial supporting a change in glaucoma care, recommending that SLT can be offered as a first-line treatment in OAG and OHT [3] In this trial of treatment-naïve patients, 74.2% of those randomised to SLT remained drop-free at 3 years [3] At 2 months following the initial SLT procedure, there was a mean IOP reduction of 8 mmHg in OHT eyes and 6.5 mmHg in OAG eyes, representing a percentage IOP reduction of 29.7% and 26.1% respectively [4] Higher baseline IOP was a predictor for a greater IOP reduction in both OAG and OHT eyes (p < 0.001) [4].

It is also important to determine the real-world outcomes of SLT, in particular as the majority patients in glaucoma clinics are not treatment-naïve, and may have been using topical IOP-lowering medications for a number of years. Real-world studies have demonstrated significant IOP reductions with SLT, although somewhat more modest than in clinical trials [5, 6].

The purpose of this study was to evaluate the real-world treatment outcomes of SLT in a tertiary referral glaucoma service.

## Methods

We reviewed the medical records of consecutive patients who had undergone SLT in the glaucoma service at the Royal Victoria Eye and Ear Hospital in Dublin, Ireland. All laser procedures were performed by one ophthalmologist, between 1st August 2022 and 30th June 2023. All patients in this study were using topical IOP-lowering medications prior to SLT, and continued using them thereafter.

Goldmann applanation tonometry was used to obtain IOP measurements at all points in this study. The baseline IOP was determined using the mean of two IOP measurements prior to SLT—one at the time the decision was made to proceed with SLT and the other prior to performing SLT of the day of attendance for the procedure. In all cases, SLT was performed within 120 days of the decision to proceed. The cohort did not include any patients with a history of uveitis, previous glaucoma surgery, angle closure, or neovascular glaucoma. The post-SLT IOP was measured at 6 to 8 weeks following the procedure. Other baseline data included age, gender, diagnosis, concurrent use of topical IOP-lowering medication and previous SLT.

SLT was performed in a standard manner, by performing 100 non-overlapping laser shots to 360° of the trabecular meshwork using an appropriate SLT lens, following the protocol as described previously in the LiGHT trial [3, 7] Laser energy varied from 0.3 to 1.4 mJ and was titrated throughout the procedure to achieve fine “champagne bubbles” 50% of the time [3, 7] A topical anti-cholinergic miotic agent (pilocarpine 2%) and α-agonist (apraclonidine 0.5%) were administered prior to the procedure. IOP was checked 1 h following the procedure. A topical non-steroid anti-inflammatory drop (ketorolac 0.5%) was prescribed 3 times a day for 5 days after SLT, to be used only if patients experienced ocular discomfort symptoms.

The primary outcome measure was the mean change in intraocular pressure (IOP) at 6 to 8 weeks following the procedure. Statistical analysis was performed to assess predictors of greater IOP reduction following SLT [8].

## Results

71 eyes of 44 patients were included in this study. Baseline characteristics are demonstrated in Table 1. The mean age at the time of SLT was 72.2 years. 59.1% of the cohort was male. The diagnosis was primary open-angle glaucoma (POAG) in 69.0% of eyes, pseudoexfoliation glaucoma (PXFG) in 5.6%, pigment dispersion glaucoma (PDG) in 2.8%, normal tension glaucoma (NTG) in 5.6%, ocular hypertension (OHT) in 15.5%, and pseudoexfoliation OHT in 1.4%. 100% of eyes (n = 71) were on topical IOP-lowering medication (mean number of agents 2.7). 8.5% of eyes had undergone one previous SLT.

**Table 1:** Baseline characteristics

	n = 71 eyes (44 patients)
Age, mean (years)	72.2 years
Gender Male Female	59.1% 40.9%
Baseline IOP, mean (SD) (mmHg)	19.1 (3.6)
Diagnosis (% of eyes) POAG PXFG PDG NTG OHT PXF OHT	69.0% 5.6% 2.8% 5.6% 15.5% 1.4%
Treatment (% of eyes) Topical IOP-lowering medication Prostaglandin analogue β-blocker Carbonic anhydrase inhibitor α-agonist Previous SLT	100% 98.6% 69.0% 77.5% 21.1% 8.5%

POAG = primary open angle glaucoma. PXFG = pseudoexfoliation glaucoma. PDG = pigment dispersion glaucoma. NTG = normal tension glaucoma. OHT = ocular hypertension. PXF OHT = pseudoexfoliation ocular hypertension

There was a mean reduction in IOP of 4.0 mmHg (SD 3.6), from 19.1 mmHg (SD 3.6) at baseline to 15.1 mmHg (SD 4.0) at 6 to 8 weeks post-SLT (p < 0.001). The assumption of normality was confirmed (Shapiro–Wilk test: p = 0.142), and a subsequent paired sample t-test indicated that this was a statistically significant change (t = 9.455, p < 0.001).

This represents a 20.9% reduction in IOP across the whole cohort. 77.5% of eyes had at least a 10% reduction in IOP, while 39.4% of eyes had at least a 25% reduction.

Eyes with a diagnosis of OHT (n = 11) had a mean IOP reduction of 4.1 mmHg, from 21.4 mmHg at baseline to 17.3 mmHg after SLT. Eyes with OAG (POAG, PDG or PXFG, n = 55) also had a mean IOP reduction of 4.1 mmHg, from 19.0 mmHg at baseline to 14.9 mmHg after SLT. Eyes with NTG (n = 4) had a mean IOP reduction of 2.6 mmHg, from 13.6 mmHg at baseline to 11.0 mmHg. Eyes with pseudoexfoliation (PXFG or PXF OHT, n = 5) had a mean IOP reduction of 1.8 mmHg, from 18.6 mmHg at baseline to 16.8 mmHg. Eyes that had previous SLT (n = 6) had a mean IOP reduction of 4.5 mmHg, from 22.5 mmHg at baseline to 18.0 mmHg.

**Fig. 1:**
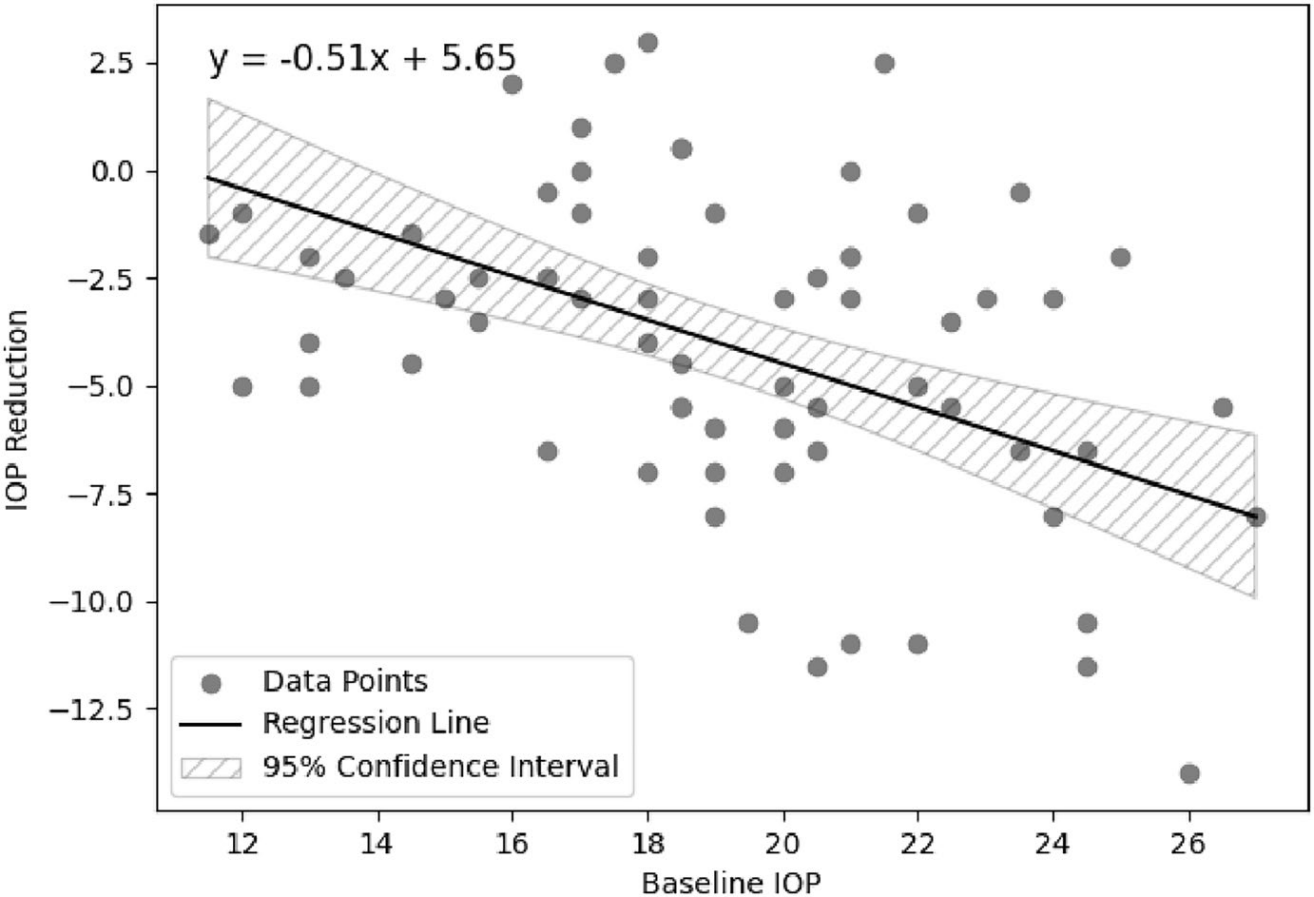
Regression line depicting the relationship between baseline IOP (mmHg) and IOP reduction (mmHg) across the study population, based on fixed effects estimates from a mixed effects model

Pearson correlation analysis revealed a moderate correlation between higher baseline IOP and greater IOP reduction(r = − 0.4021, p < 0.001). As the data was from a sample of 71 eyes of 44 distinct patients, further analysis was performed using a mixed effects linear regression model to account for the interocular correlation of measurements among patients. The mixed effects model, estimated via restricted maximum likelihood (REML), also showed a significant correlation between higher baseline IOP and greater IOP reduction (β = − 0.51, SE = 0.122, z = − 4.162, p < 0.001), indicating a 0.51-unit increase in IOP reduction for each unit increase in baseline IOP, accounting for patient variability. A regression line derived from the fixed effects estimates of the model (Fig. 1) visually represents the relationship between baseline IOP and IOP reduction across the study population. Inter-patient variance was 9.630 (SE = 2.708), highlighting the variability in IOP reduction outcomes among patients, and also justifying our use of the mixed effects model. Other parameters including gender, previous SLT, lower number of IOP-lowering agents, and the presence of pseudoexfoliation did not demonstrate a significant correlation with IOP change.

No immediate (< 1 h) post-SLT IOP spikes (> 5 mmHg) occurred in this study. In addition, no cases of post-SLT anterior chamber inflammation were identified.

## Discussion

This real-world study supports the use of SLT as an adjunctive therapy in patients with open-angle glaucoma and ocular hypertension, who are already using IOP-lowering topical medication. Our cohort achieved a mean IOP reduction of 4.0 mmHg (SD 3.6), from 19.1 mmHg (SD 3.6) at baseline to 15.1 mmHg (SD 4.0) at 6 to 8 weeks post-SLT (p < 0.001). This represents a 20.9% reduction in IOP across the study population.

As expected, this is a more modest IOP reduction when compared to clinical trials of treatment-naïve patients, with the LiGHT trial demonstrating a mean IOP reduction of 8 mmHg in OHT eyes and 6.5 mmHg in OAG eyes at 2 months after SLT [4] However our results are comparable to other real-world studies in which the majority of patients were already using IOP-lowering medication [5, 6] A 2020 real-world study of 831 patients, 37% of whom were treatment-naïve, demonstrated a mean IOP reduction of 4.2 mmHg at 12 to 18 months after SLT, from 22 mmHg at baseline (p < 0.0001) [5] Similarly, a 2022 real-world study of 835 patients, 16.1% of whom were treatment-naïve, demonstrated a mean IOP reduction of 3.6 mmHg after SLT, from 18.4 mmHg at baseline (p < 0.001) [6].

In our study, there was a significant correlation between higher IOP and a greater IOP reduction. This was found to be significant on Pearson correlation analysis and a mixed effects linear regression model to account for interocular correlation, as both eyes of some patients were included [8] This is in keeping with the previous findings among treatment-naïve patients during the LiGHT trial, which demonstrated that higher baseline IOP was associated with greater absolute IOP-lowering at 2 months after primary SLT [9] In our cohort, the mixed effects model quantified the correlation between higher baseline IOP and greater IOP reduction (β = − 0.51, SE = 0.122, z = − 4.162, p < 0.001), indicating that for each unit increase in baseline IOP there was a 0.51-unit increase in IOP reduction.

Other parameters including gender, previous SLT, lower number of IOP-lowering agents, and the presence of pseudoexfoliation did not demonstrate a significant correlation with IOP change after SLT, however this is of uncertain significance considering our relatively small cohort. It has previously been suggested that female gender and previous SLT are associated with less absolute IOP reductions at 2 months after SLT [9, 10].

No post-SLT IOP spikes (> 5mm Hg) or anterior chamber inflammation occurred in this study. This is consistent with previous evidence, demonstrating SLT to have a good safety profile [3] Among the 356 patients treated with SLT in the LiGHT trial, 0.3% (n = 1) had post-laser inflammation, while 1.7% (n = 6) demonstrated post-laser IOP spikes (> 5 mmHg) with just one of those patients requiring IOP-lowering treatment [3].

This study has some limitations. It is a retrospective review using data collected in real-world practice. It is also a relatively small sample size with a short duration of follow-up. However, our study also has multiple strengths. All patients were treated with a standardised SLT protocol, performed by a single ophthalmologist. Furthermore, all patients were already on IOP-lowering drops, which is reflective of real-world practice. As such, it does provide useful evidence on the short term efficacy of SLT as an adjunctive therapy.

In conclusion, SLT is an effective IOP-lowering treatment in open-angle glaucoma and ocular hypertension in a real-world setting.

## Data Availability

No datasets were generated or analysed during the current study.
